# Protein and calorie restriction may improve outcomes in living kidney donors and kidney transplant recipients

**DOI:** 10.18632/aging.103619

**Published:** 2020-07-11

**Authors:** Franny Jongbloed, Ron W.F. de Bruin, Harry Van Steeg, Piet Beekhof, Paul Wackers, Dennis A. Hesselink, Jan H.J. Hoeijmakers, Martijn E.T. Dollé, Jan N.M. IJzermans

**Affiliations:** 1 Department of Surgery, Erasmus MC, University Medical Center Rotterdam, Rotterdam, The Netherlands; 2 Laboratory of Health Protection Research, National Institute of Public Health and the Environment, Bilthoven, The Netherlands; 3 Department of Toxicogenetics, Leiden University Medical Center, Leiden, The Netherlands; 4 Department of Internal Medicine, Erasmus MC, University Medical Center Rotterdam, Rotterdam, The Netherlands; 5 Department of Genetics, Erasmus MC, University Medical Center Rotterdam, Rotterdam, The Netherlands

**Keywords:** dietary restriction, living kidney donation, kidney transplantation, kidney function, acute rejection

## Abstract

Previously, we and others showed that dietary restriction protects against renal ischemia-reperfusion injury in animals. However, clinical translation of preoperative diets is scarce, and in the setting of kidney transplantation these data are lacking. In this pilot study, we investigated the effects of five days of a preoperative protein and caloric dietary restriction (PCR) diet in living kidney donors on the perioperative effects in donors, recipients and transplanted kidneys. Thirty-five kidney donors were randomized into either the PCR, 30% calorie and 80% protein reduction, or control group without restrictions. Adherence to the diet and kidney function in donors and their kidney recipients were analyzed. Perioperative kidney biopsies were taken in a selected group of transplanted kidneys for gene expression analysis. All donors adhered to the diet. From postoperative day 2 up until month 1, kidney function of donors was significantly better in the PCR-group. PCR-donor kidney recipients showed significantly improved kidney function and lower incidence of slow graft function and acute rejection. PCR inhibited cellular immune response pathways and activated stress-resistance signaling. These observations are the first to show that preoperative dietary restriction induces postoperative recovery benefits in humans and may be beneficial in clinical settings involving ischemia-reperfusion injury.

## INTRODUCTION

Dietary restriction (DR) increases resistance to oxidative stress, including ischemia-reperfusion injury (IRI) [1–4]. IRI arises from acute oxidative stress that inevitably occurs during kidney transplantation [5, 6]. Although living donor kidney transplantation greatly improves function and survival of kidney allografts compared to kidneys from deceased donors [7], IRI remains a risk factor for poor transplant outcome [8–10]. We demonstrated that protection against renal IRI is induced by preoperative fasting, DR and by dietary deprivation of protein alone, indicating that the effects of calorie and protein restriction might act synergistically [11–13]. Translation of the beneficial effects of short-term DR to humans has proven difficult and unsuccessful [14]. Previously, we reported the results of a randomized controlled clinical trial which demonstrated that a diet combining calorie and protein restriction (PCR) diet is feasible and safe in living kidney donors, as well as in patients undergoing bariatric surgery [15]. We performed this pilot study to investigate the efficacy of PCR by examining the perioperative and postoperative effects in living kidney donors, their recipients and the transplanted kidneys.

## RESULTS

### Baseline characteristics

Thirty-five living kidney donors were randomized into either the PCR (n=15) or the control group (n=20) between May 2, 2014 and November 18, 2015 (Supplementary Figure 1). The difference in patient numbers between the groups is due to the additional inclusions to replace dropouts. Baseline characteristics of these 35 donors are listed in Table 1A. Donors in the PCR-group were more often female (*P*=0.028) and consequently had lower serum creatinine concentrations (*P*=0.021). Baseline characteristics of all transplant recipients (Table 1B) showed no differences between the two groups.

**Table 1A:** Baseline characteristics of living kidney donors prior to the start dietary intervention.

**Parameter**	**PCR (n=15)**	**Control (n=20)**	**P-value**
Age (years)	55 (51-55)	54 (46-59)	0.395
Gender (Male/Female)	4/11	13/7	**0.028**
BMI (kg/m^2^)	23.7 (22.4-27.6)	26.0 (26.6-29.1)	0.157
Systolic blood pressure (mm/Hg)	128 (123-137)	128 (122-137)	1.000
Creatinine (mmol/L)^#^	71 (66-78)	80 (71-91)	**0.021**
CKD-EPI eGFR (mL/min)	86 (72-90)	80 (73-89)	0.672
Urea (mmol/L)	5.3 (4.4-5.8)	5.3 (4.2-6.1)	0.986
Glucose (mmol/L)	5.3 (5.0-5.7)	5.0 (4.8-5.9)	0.217
Albumin (g/L)	47 (44-48)	46 (45-47)	0.398
Triglycerides (mmol/L)	1.22 (0.85-1.50)	1.31 (1.00-1.68)	0.488
Hemoglobin (mmol/L)^#^	8.8 (8.4-9.0)	9.2 (8.7-9.6)	**0.031**
Trombocytes (10^9^/L)	234 (213-294)	253 (206-289)	0.972
CRP (mg/L)	1.1 (0.7-1.9)	1.5 (0.6-2.4)	0.259
Leukocytes (10^9^/L)	6.6 (5.5-7.4)	6.6 (5.5-7.8)	0.652
Bilirubin (μmol/L)	6 (5-10)	7 (5-12)	0.467
Potassium (mmol/L)	4.4 (4.2-4.6)	4.3 (4.1-4.7)	0.444
Type of donation (R/U/A)	3/3/9	4/12/4	0.083
Side of nephrectomy (Left/Right)	8/7	12/8	0.712
Method used (Laparoscopic/HARP)	12/3	14/6	0.523

**Table 1B:** Baseline characteristics of kidney transplant recipients prior to surgery.

**Parameter**	**PCR (n=15)**	**Control (n=20)**	**P-value**
Age (years)	56 (44-67)	54 (45-58)	0.250
Gender (Male/Female)	8/7	10/10	0.863
Kreatinine (mmol/L)	495 (442-637)	449 (321-902)	0.351
CKD-EPI eGFR (mL/min)*	9 (7-11)	11 (5-14)	0.471
Urea (mmol/L)	19.9 (16.2-31.4)	22.6 (15.0-27.1)	0.881
Potassium (mmol/L)	5.4 (4.7-5.8)	4.7 (4.4-5.3)	0.092
Hemoglobin (mmol/L)	6.6 (6.2-7.5)	7.3 (6.6-8.3)	0.166
CRP (mg/L)	2.7 (1.5-4.7)	2.1 (0.6-5.2)	0.205
Leukocytes (10^9^/L)	7.8 (6.8-9.7)	6.1 (5.5-8.2)	0.106
Type of donation (R/U/A)	3/3/9	4/12/4	0.083
Immunosuppressive therapy prior to transplantation (Yes/No)	4/11	5/15	1.000
Dialysis prior to transplantation (Yes/No)	6/9	9/11	0.687
Side of transplantation (Left/Right)	4/11	7/12	0.549

Values are depicted as median ± interquartile range. Significant values are depicted in bold. BMI = body mass index; CKD-EPI eGFR = estimated glomerular filtration rate using the CKD-EPI formula; CRP = C-reactive protein. Type of donation: R = related; U = unrelated; A = anonymous. HARP = hand-assisted retroperitoneal nephrectomy. ^#^= both creatinine and hemoglobin levels of the living kidney donors were lower in the PCR than the control group as a consequence of the gender differences in both groups. * = all kidney transplant recipients met the criteria for end-stage renal disease with a CKD-EPI eGFR <15 mL/min. Significance = *P*<0.05.

### Compliance and perioperative outcome

All PCR-donors indicated that they had adhered to the diet. This was in line with a significant decrease of serum levels of prealbumin (PAB), retinol binding protein (RBP), valine and leucine after the PCR diet (Supplementary Figure 1). PCR-donors lost an average of 2.0 kg (range -4;0 kg) of bodyweight during the diet, while the control group gained 0.4 kg (range -4; +1 kg) (*P*=0.006). Perioperative outcomes did not significantly differ between the two arms in both donors and recipients (Supplementary Table 1A).

### Postoperative outcome

### Donors

**Figure 1:**
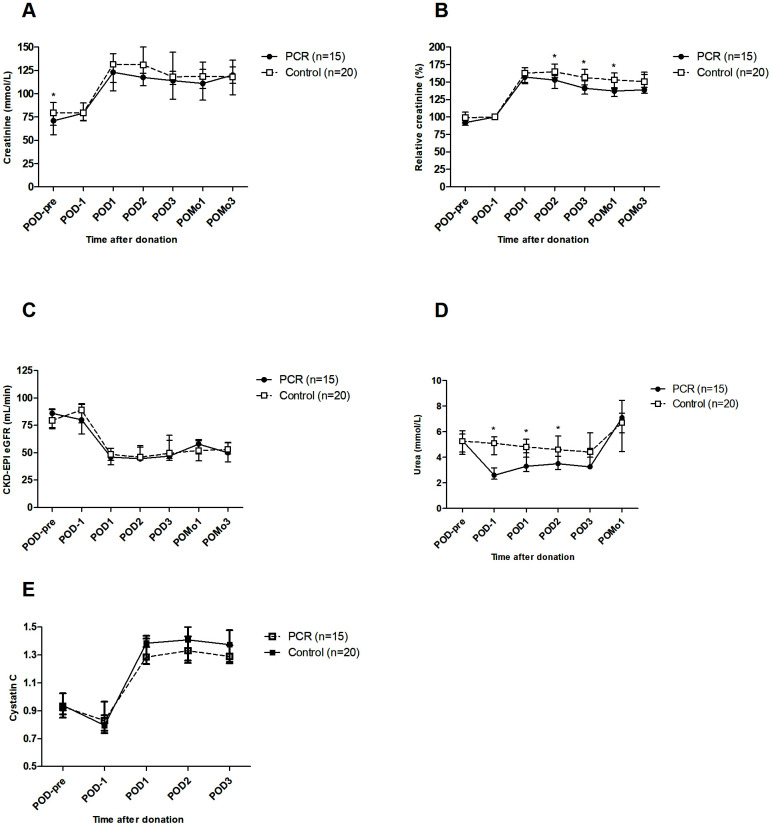
**Kidney function of living kidney donors before and after kidney donation.** (**A**) At start of the study (POD-pre), creatinine levels were significantly higher in the control group compared to the PCR group. Postoperatively, a trend towards lower levels of creatinine was observed in the PCR-group. (**B**) Taking POD-1 as cut-off value, relative creatinine clearance was significantly improved in the PCR-group at POD2, POD3 and POMo1. (**C**) Absolute glomerular filtration rate did not significantly differ between the groups. (**D**) Serum urea levels were significantly lower in the PCR group on POD1 and remained so in the first two postoperative days. (**E**) Serum Cystatin C concentrations showed no significant differences between the two groups except for a trend of lowering values in PCR-donors on POD3. Values are depicted as median ± interquartile range. PCR = protein and caloric dietary restriction; POD = postoperative day; POMo = postoperative month; eGFR = estimated glomerular filtration rate using the CKD-EPI formula. *=significant values.

At baseline, serum creatinine concentrations significantly differed between donors in the PCR and the control group (Figure 1A, postoperative day (POD)-pre). One day before surgery (*i.e.* at day 5 of dietary intervention), serum creatinine concentrations were similar (Figure 1A, POD-1). In a linear mixed-effects model analysis, PCR-donors showed significantly lower creatinine concentrations than the control group on POD2, POD3 and on postoperative month (POMo)1 (Supplementary Table 2A). Absolute CKD-EPI estimated glomerular filtration rate (eGFR) values did not significantly differ in both groups. To correct for baseline interpatient variability, the relative values of creatinine were calculated and analyzed. Corresponding to the absolute concentrations, relative creatinine concentrations were significantly improved in the PCR-donors on POD2, POD3 and POMo1 (Supplementary Table 2A). Subsequently, changes per time point were assessed. The PCR-donors showed a trend towards better absolute creatinine concentrations (Figure 1A); Relative creatinine concentrations were significantly better on POD2 (*P*=0.030), POD3 (*P*=0.042) and POMo1 (*P*=0.040, Figure 1B). Absolute eGFR values did not differ (Figure 1C). Serum urea levels were significantly lower in the PCR-group on POD-1 (*P*<0.001), suggesting effects of the PCR-diet on kidney function *per se*. This difference persisted after surgery on POD1 (*P*=0.003), POD2 (*P*=0.005) and diminished on POD3 (*P=*0.09) (Figure 1D). Cystatin C, as a marker of kidney function independent of muscle mass, showed no significant difference before or after the PCR-diet nor postoperatively; however, concentrations of Cystatin C did show on average a decreasing trend in PCR-donors on POD3 compared to controls (*P*=0.062, Figure 1E).

**Figure 2:**
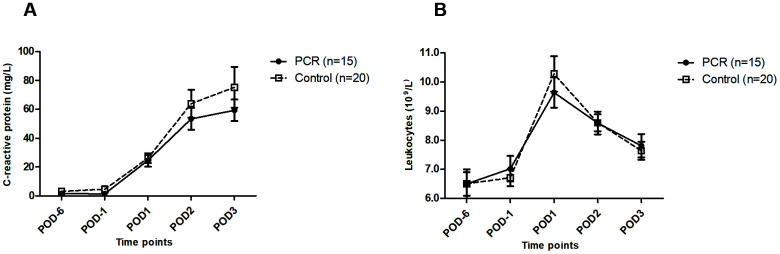
**Systemic inflammatory markers in living kidney donors before and after kidney donation.** (**A**) Levels of systemic inflammatory marker C-reactive protein before and after live kidney donation were not significantly different between the PCR and the control group. (**B**) Levels of leukocytes did not significantly differ between both groups either, and only reached high-normal levels on POD1 after surgery. Values are depicted as median ± interquartile range. PCR = protein and caloric dietary restriction; POD = postoperative day.

Postoperative C-reactive protein (CRP) concentrations showed a trend towards lower levels in the PCR-donors, with a broad interquartile range (IQR) (Figure 2A). No differences in leukocyte numbers were seen (Figure 2B).

### Recipients

Ideally, urine production in kidney transplant recipients starts directly after revascularization of the graft. In the PCR-group, urine production was delayed in 1/15 (7%) patients compared with 5/20 (25%) in the control group (*P*=0.135) (Table 2 and Supplementary Table 1B). On POD1, a trend towards lower incidence of partial acute tubular necrosis (ATN), as indicated on a MAG3 scan, was found in the PCR-group (Table 2). Delayed graft function (DGF) did not occur. Slow graft function (SGF) occurred significantly more often in the control group: 5/20 (25%) *versus* 0/15 (0%) (*P*=0.020). The incidence of biopsy-proven acute rejection (BPAR) was significantly higher in the control group than in the PCR-group: 8/20 (40%) *versus* 1/15 (7%) (*P*=0.013). Two recipients (both in the control group) developed severe acute rejection and underwent transplant nephrectomy on days six and 12 after transplantation, respectively. Data from these patients were censored from the day of transplant removal. The postoperative immunosuppressive drug regimen was similar in both groups (tacrolimus (Tac), mycophenolate mofetil (MMF) and prednisolone), except for two patients in the control group who received belatacept instead of Tac. These two patients experienced BPAR and were switched from belatacept to Tac. The Tac pre-dose concentrations showed a trend towards higher concentrations in the PCR-group on POD3 (*P*=0.065) and POD5 (*P*=0.094). No significant differences were seen in the duration of hospital stay nor in the incidence or severity of postoperative complications (Table 2).

**Table 2:** Postoperative outcome and complications in kidney transplant recipients in the first 14 days after surgery.

**Parameter**	**PCR (n=15)**	**Control (n=20)**	***P*-value**
Urine production during surgery (%)	1/15 (7%)	5/20 (25%)	0.135
ATN on MAG3 scan (%)	2/15 (7%)	6/20 (30%)	0.209
Delayed graft function (%)	0/15 (0%)	0/20 (0%)	1.000
Slow graft function (%)	0/15 (0%)	5/20 (25%)	**0.020**
Acute rejection, biopsy-proven (%)	1/15 (7%)	8/20 (40%)	**0.013**
Hospital stay (days)	13 ± 1	14 ± 1	0.556
Any complication (yes/no)	10/5 (73%)	16/4 (75%)	0.445
Clavien-Dindo score (0-4)	1.5 ± 0.3	1.5 ±0.2	0.986
Tacrolimus level POD3	15.0 ± 1.8	10.6 ± 1.4	0.065
Tacrolimus level POD5	14.5 ± 1.8	10.8 ± 1.2	0.094
Tacrolimus level POD10 (μg/L)	12.7 ± 1.5	10.6 ± 1.0	0.251

Values are depicted as median ± interquartile range. ATN = acute tubular necrosis; MAG3 = renal scintigraphy scan; POD = postoperative day; Clavien-Dindo = official classification score for postoperative surgical complications. PCR = protein and caloric dietary restriction. Significance is considered at *P*<0.05 and is depicted in bold.

**Figure 3:**
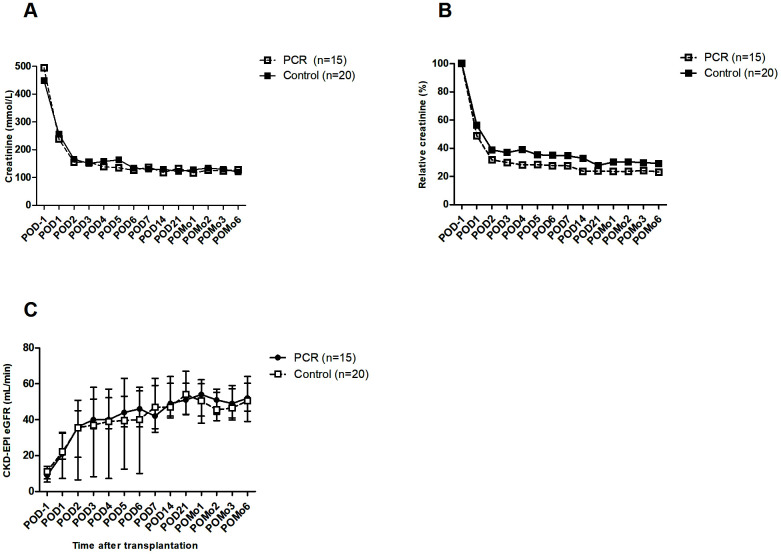
**Kidney function of transplant recipients before and after surgery.** (**A**) Serum creatinine showed a trend towards absolute lower levels in the PCR-group throughout the first 14 days after surgery. (**B**) Relative creatinine levels, calculated using POD-1 as cutoff value, showed a trend as from POD4 up to POD14. (**C**) Following the creatinine clearance, eGFR also showed a trend towards improvement in the PCR-group throughout the first days after surgery. Values are depicted as median ± interquartile range. PCR = protein and caloric dietary restriction; POD = postoperative day; POMo = postoperative month; eGFR = estimated glomerular filtration rate using the CKD-EPI formula.

Absolute and relative creatinine concentrations were significantly improved in the PCR-recipients as from POD1 until POD5 (Supplementary Table 2B). Assessment per time point showed no significant differences of absolute serum creatinine concentrations between groups throughout the postoperative follow-up (Figure 3A). Relative creatinine concentrations showed a trend towards lower concentrations in the PCR-group on POD4 (*P*=0.057), POD6, and POD14 (Figure 3B). Absolute eGFR values did not differ between groups (Figure 3C).

### Gender-based differences

Since gender was unevenly distributed between the groups, a gender stratified analysis was performed. In female donors, a consistent trend towards improved relative creatinine concentrations was observed for prolonged periods after POD1 (Supplementary Figure 2). In male donors, relative creatinine concentrations were significantly improved in the PCR-group at POMo1 (*P*=0.009) and POMo3 (*P*=0.022*)* (Supplementary Figure 3). Division based on gender of the donors showed for female PCR-kidney recipients a consistent non-significant trend towards improved relative creatinine concentrations (Supplementary Figure 2E–2G). The most pronounced PCR-related differences in the recipients were observed in kidneys of male donors from POD1 until POD7, yet did not reach significance due to large variation (Supplementary Figure 3E–3G).

### Renal transcriptome analysis

**Figure 4:**
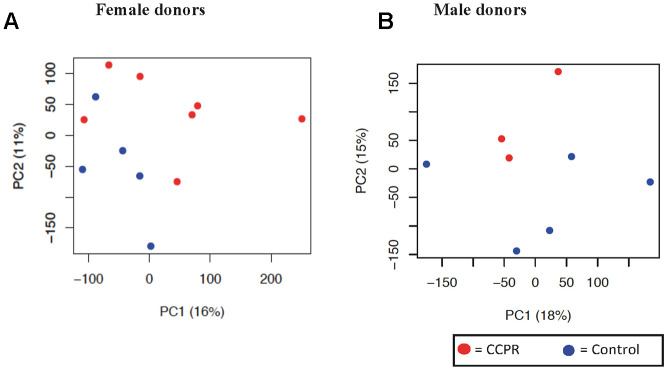
**Principal component analysis of kidney tissue in female and male donors.** (**A**) Unbiased principal component analysis (PCA) of all female donors showed the most variation of genes on the principal component (PC) axis 1 and some clustering of the two intervention groups is shown. (**B**) The PCA of the male donors was based on only three kidneys in the PCR group, and showed little variation on PC axis 2. Principal component (PC) 1 is depicted on the x-axis and PC2 is depicted on the y-axis, followed by the percentage of variance explained by each axis. Each symbol represents one sample of one donor. Samples of the same group are shown in the same color. PCR = protein and caloric dietary restriction.

Full genome expression profile analysis of the kidneys was performed using biopsies from 20 donors (Supplementary Table 3). A principal component analysis (PCA) showed that gender represented the largest discriminator (Supplementary Figure 4). Therefore, we separately analyzed male (n=3-5) and female (n=5-7) kidneys. The PCAs of both female (Figure 4A) and male (Figure 4B) kidney transcriptome showed small inter-variability on PC axis 1 and 2,

respectively. Since one sample had to be excluded due to a low RNA yield, only 3 male patients in the PCR-group remained. Therefore, statistical analysis was restricted to the female data, showing 480 differentially expressed transcripts (DET) between diet groups. Pathway analysis indicated *EIF2 Signaling* as significantly upregulated (Table 3A). Other importantly enriched and activated pathways included the NRF2-pathway, G2/M checkpoint regulation and amino acid metabolism. Three significantly activated – MYCN, MYC and NKX2-3 – and two significantly inhibited – PRDM1 and SMARCB1 – transcription factors were found (Table 3B). The pathway analysis done in the male donors can be found in Supplementary Table 4.

**Table 3A:** Pathway analysis in female kidney tissue.

**Ingenuity canonical pathways**	**Ratio**	**Up/Down**	***P*-value**	**Z-score**
**eIF2 Signaling**	**9/221 (4.1%)**	**9/0**	**6.50^E^-04**	**2,449**
Primary Immunodeficiency Signaling	4/48 (8.3%)	0/4	1.72^E^-03	N/A
Interferon Signaling	3/36 (8.3%)	0/3	6.65^E^-03	N/A
Complement System	3/37 (8.1%)	0/3	7.18^E^-03	N/A
Aldosterone Signaling in Epithelial Cells	6/166 (3.6%)	5/1	9.03^E^-03	N/A
Autoimmune Thyroid Disease Signaling	3/47 (6.4%)	0/3	1.39^E^-02	N/A
Hematopoiesis from Pluripotent Stem Cells	3/47 (6.4%)	0/3	1.39^E^-02	N/A
Communication between Innate and Adaptive Immune Cells	4/89 (4.5%)	0/4	1.53^E^-02	N/A
Cell Cycle: G2/M DNA Damage Checkpoint Regulation	3/49 (6.1%)	2/1	1.55^E^-02	N/A
NRF2-mediated Oxidative Stress Response	6/193 (3.1%)	6/0	1.79^E^-02	N/A
Asparagine Degradation I	1/2 (50.0%)	0/1	2.13^E^-02	N/A
β-alanine Degradation I	1/2 (50.0%)	0/1	2.13^E^-02	N/A
Proline Degradation	1/2 (50.0%)	0/1	2.13^E^-02	N/A
Alanine Degradation III	1/2 (50.0%)	0/1	2.13^E^-02	N/A
Alanine Biosynthesis II	1/2 (50.0%)	0/1	2.13^E^-02	N/A

**A.** The top 15 overrepresented pathways derived from the differentially expressed transcripts (DET) in the PCR diet compared to the control group in kidney tissue of female donors. The pathways show their corresponding ratio of regulated genes as percentage of total genes in the pathway, the *P*-value and the Z-score for predicted activation or inhibition of the pathways. Significantly activated pathway is depicted in bold.

**Table 3B:** Upstream transcription factor analysis in female kidney tissue.

**Upstream regulator**	**Description**	**Z-score**	***P*-value**	**Gene log ratio**
**MYCN**	MYCN proto-oncogene, bHLH transcription factor	**+2.985**	8.83^E^-04	-0.102
**MYC**	MYCN proto-oncogene, bHLH transcription factor	**+2.191**	1.00 ^E^-00	-0.325
**NKX2-3**	NK2 homeobox 3	**+2.000**	2.07 ^E^-01	0.197
NKFBIA	NFκB inhibitor alpha	+1.492	4.64 ^E^-02	-0.349
SMARCA4	SWI/SNF related, matrix associated, actin dependent regulator of chromatin, subfamily a, member 4	+0.600	1.44 ^E^-02	-0.387
MITF	Melanogenesis associated transcription factor	+ 0.447	4.31 ^E^-02	0.322
STAT3	Signal transducer and activator of transcription 3	+ 0.391	7.60 ^E^-04	-0.433
PITX2	Paired like homeodomain 2	+ 1.065	2.23 ^E^-03	-0.065
SATB1	SATB homeobox 1	+0.128	8.67 ^E^-03	0.365
WT1	Wilms tumor 1	+ 0.101	4.24 ^E^-02	0.265
HNF4A	Hepatocyte nuclear factor 4 alpha	-0.315	7.41 ^E^-03	-0.239
HSF1	Heat shock transcription factor 1	-0.251	4.50 ^E^-02	-0.271
GATA3	GATA binding protein 3	-0.425	2.77 ^E^-02	-0.360
KMT2D	Lysine methyltransferase 2D	-0.447	3.99 ^E^-02	-0.251
EGR1	Early growth response 1	-0.600	4.68 ^E^-02	0.130
TCF7L2	Transcription factor 7 like 2	-0.632	2.99 ^E^-03	-0.329
PML	Promyelocytic leukemia	-0.785	4.28 ^E^-04	-0.558
SMAD4	SMAD family member 4	-1.067	1.21 ^E^-02	-0.361
STAT1	Signal transducer and activator of transcription 1	-1.534	1.23 ^E^-02	-0.431
CREB1	cAMP responsive element binding protein 1	-1.591	1.97 ^E^-02	0.295
IRF7	Interferon regulatory factor 7	-1.671	8.91 ^E^-03	-0.374
BRCA1	BRCA1, DNA repair associated	-1.719	1.24 ^E^-04	0.228
POU2AF1	POU class 2 associating factor 1	-1.987	2.87 ^E^-03	+0.287
**PRDM1**	PR/SET domain 1	**-2.121**	2.75 ^E^-03	-0.429
**SMARCB1**	SWI/SNF related, matrix associated, actin dependent regulator of chromatin, subfamily b, member 1	**-2.236**	9.96 ^E^-03	0.351

**B**. Differentially regulated upstream transcription factors (TFs) derived from the DET in the PCR diet compared to the control group in kidney tissue of female donors, with their corresponding Z-score, *P*-value and gene log ratio. Significantly activated or inhibited TFs are depicted in bold.

## DISCUSSION

Animal studies have unequivocally established the beneficial effects of short-term DR regimens, including fasting, calorie-, and protein restriction, on renal and hepatic IRI [11–13, 16]. Translation to clinical interventions has proven difficult because of concerns about the possible side effects of preoperative restrictive diets, lack of voluntary adherence and uncertainty regarding the DR strategy to be used [14]. Our randomized, controlled clinical pilot study demonstrates that five days of PCR before kidney donation is feasible, safe and suggests improvement of renal recovery in both donors and recipients, and reduction of the incidence of slow graft function and acute rejection.

The fact that all 15 donors adhered to the diet, shown by weight loss and compliance markers in serum, confirmed our previous observation that PCR is feasible. Levels of systemic postoperative inflammation markers were similar in both donor groups, suggesting the PCR-diet does not affect the postoperative systemic inflammatory response [14]. The incidence of perioperative and postoperative complications in the donors was also similar between groups, underscoring the safety of the PCR-diet and showing that concerns regarding a compromised immune response and wound healing following DR in a surgical setting are unwarranted [14].

The protective effects of the PCR-diet on the kidney were shown both pre- and postoperatively by significantly improved serum urea and creatinine concentrations, and eGFR. These differences in creatinine and urea levels observed between groups is probably due to the decreased production of waste product resulting in a lower rate of removal by the kidneys. The improved postoperative kidney function in donors in a setting of only marginal decrease in creatinine concentrations due to kidney removal itself [17], further underscores the robust effects of the PCR-diet. Subgroup analyses based on gender showed an improved renal outcome in both male and female donors. The ameliorated outcome in the donors is further strengthened by the significantly, clinically highly relevant improvement in creatinine concentrations in the kidney transplant recipients from PCR-donors. Transplant recipients of PCR-donors showed a trend towards higher Tacrolimus pre-dose concentrations which suggests PCR alters the metabolism of Tacrolimus. Although higher exposure to Tac is associated with increased nephrotoxicity [18], the creatinine concentrations were better in the PCR-recipients. Together with the significantly lower incidence of acute rejection and SGF, these data indicate that PCR induces increased stress resistance in humans resulting in protection of the transplanted kidney from ischemic damage and from acute rejection. Two recipients in the control group developed uncontrollable acute rejection which necessitated transplant nephrectomy [19].

However, these patients received belatacept rather than Tac-based immunosuppression. Since belatacept is less potent than Tac, we feel that these two cases of severe rejection are explained by the immunosuppressive therapy [20]. These patients were excluded from analyses from the day of transplant removal and could not further influence the outcome.

Transcriptome analysis in kidney tissue obtained during surgery revealed upregulation of NRF2-mediated stress response and eIF2 signaling and inhibition of cell cycle G2M phase regulation due to the PCR-diet. These data strongly overlap with our data and data from others in kidneys of mice subjected to protein-free, calorie-restricted or fasting diets [11, 12, 21]. Activation of eIF2 reduces global translation, allowing cells to switch from growth to maintenance and inducing stress resistance [22]. Together with the upregulation of NRF2, these similarities highlight the evolutionary conservation of the response to DR, which is already present in human kidneys after five days of PCR. In addition, a trend towards inhibition of the immune response was seen. Other studies using DR in heart, skeletal muscle and liver in animal models showed similar effects on cell cycle regulation and immune system signaling as well as an upregulation the NRF2 pathway [23–26]. Recently, the transcription factor *Myc* was found as an important signaling molecule activated by protein restriction in *Drosophila*, potentiating innate immunity and increasing stress resistance [27]. We found Myc as one of the highest activated transcription factors following PCR. Since pharmacological overexpression of *Myc* mimicked the effect of DR, *Myc* might well be a target to develop a DR-mimetic [27]. A transcriptome profiling study in human kidneys three months after transplantation identified downregulation of ankyrin repeat and SOCS Box containing 15 (*ASB15)* to be correlated to worse allograft outcome at 12-months post-transplantation [28]. *ASB15* was upregulated in the PCR-group in our unbiased profiling, suggesting a role in the protection against acute and chronic rejection [29, 30]. Another study, examining the link between interstitial fibrosis and gene expression profiles at 12 months after transplantation [31] found a relationship between increased serum levels of RBP and worse creatinine clearance. As we found decreased RBP levels due to the PCR-diet, it might be worth looking into the functional role of RBP in relation to renal outcome. Taken together, transcriptome analysis suggests the upregulation of stress resistance pathways and downregulation of the immune response by PCR.

This study has several limitations. It was designed as a pilot study in a single transplantation center, therefore only a small cohort of patients was included without prior power calculations. Replacement of donor-recipient pairs that were excluded from the waiting list for various, non-diet related, reasons (Supplementary Figure 5) was performed until the desired number of 15 donors in the PCR-group was reached. As a result, the number of donors included in the control group was higher. Although serum creatinine and estimated GFR concentrations are universally accepted to estimate kidney function in patients with and without renal disease, we are aware these factors are dependent on numerous patient characteristics such as gender and muscle mass. Another marker for kidney function is Cystatin C, and measurement of its concentrations in the living kidney donors showed no significant differences between the two groups, except for a trend towards improved concentrations on POD3 in PCR-donors. Two patients who received Belatacept developed irreversible acute rejection were excluded from the study and from the statistical analysis. This did not impact the creatinine concentrations and eGFR results in the recipients (data not shown). A significantly higher number of female than male donors was included. This was likely due to the known difference in willingness to donate between men and women [32]. Further studies should acknowledge these differences and stratify for gender beforehand. Expression analysis was only performed at mRNA level. The inclusion of other omics technologies, and expansion of the cohort may further clarify the clinical potential and improve mechanistic insights.

In conclusion, we show that a PCR-diet for five days immediately before kidney donation accelerates adaptation of kidney function of the remaining kidney and increases resistance to IRI in human kidney transplantation as evidenced by a more rapid recovery of transplant function. Transcriptional analysis suggested the upregulation of stress resistance pathways. Given its non-invasive character and safety, our PCR-diet may have great impact on increasing resistance to IRI in organ transplantation as well as other surgical-related stressors in humans.

## MATERIALS AND METHODS

### Study design

This pilot study was designed as a randomized controlled trial and was approved by the Medical Ethics Committee (METC, MEC number 2012-134) of the Erasmus MC, University Medical Center Rotterdam, the Netherlands. All subjects provided written informed consent before inclusion. The trial was registered on October 12, 2012 in the Dutch trial registry under trial code 3663 (https://www.trialregister.nl/). This manuscript was prepared in accordance with the CONSORT 2010 statement [33].

### Subjects

Living kidney donors were approached by the trial coordinator at the outpatient clinic of the department of Surgery of the Erasmus MC, University Medical Center Rotterdam, between May 2, 2014 and November 18, 2015 (Supplementary Figure 5). Inclusion criteria were age between 18-70 years old, BMI ≥ 19, no participation in another trial 30 days prior to first contact, and no known allergies to any of the ingredients of the used dietary intervention. Exclusion criteria were not meeting the inclusion criteria, or a surgery that would not take place in the Erasmus MC due to participation in the cross-over kidney donation program [15]. Randomization took place directly after informed consent. No statistical power calculation for sample size was performed due to the pilot design of the study.

### Dietary intervention

Patients in the PCR group received a diet containing 30% fewer calories and 80% fewer protein [15]. The diet was given for five consecutive days prior to surgery. The diet was based on a synthetic liquid diet as described previously [15], and was supplemented with a limited number of low-protein and protein-free products (mainly fruits and vegetables) until the desired preset individual needs were met [34] (Supplementary Table 5). The calorie- and protein-restricted powder Scandishake® Mix shakes were kindly provided by Nutricia Advanced Medical Nutrition, The Netherlands. The control group had no dietary restrictions and continued their normal diet.

### Surgical procedures

Preoperative, perioperative, and postoperative anesthetic care concerning drug administration, ventilation and fluid regimens was carried out according to our local protocols for kidney donors and kidney recipients. Donor kidneys were obtained via either a laparoscopic nephrectomy or a hand-assisted retroperitoneal nephrectomy (HARP) [35]. Kidney transplantation was performed via an open approach, and the kidney was positioned supra-inguinally on the external iliac artery and the external iliac vein. Additional informed consent was obtained from the transplant recipients to obtain biopsies of the renal cortex, which were taken at the end of the cold ischemia time (kidney off ice) using a punch biopsy with a diameter of 4 mm.

### Immunosuppressive therapy

Kidney transplant recipients received the same initial immunosuppressive therapy, except for two patients in the control group who received belatacept (Bristol-Myers Squib, NYC, NY) instead of tacrolimus. The initial immunosuppressive treatment consisted of tacrolimus (Prograft®; Astellas Pharma, Leiden, The Netherlands), mycophenolate mofetil (MMF; CellCept®; Roche Pharmaceuticals, Woerden, The Netherlands) and prednisolone treatment. All patients received induction therapy with basiliximab (Simulect®, Novartis Pharma, Arnhem, the Netherlands). The doses, whole blood or plasma target concentrations, and phasing of immunosuppressive therapy have been described elsewhere in detail [36].

### Outcome parameters

### Living kidney donors

Before and after the dietary intervention, the following data were obtained: bodyweight, age, gender, length, and systolic blood pressure. The time point of first blood withdrawal before start of the dietary intervention, POD-pre, varied from one day up to over a year. Therefore, we used POD-1 values after the dietary intervention as cutoff levels for the calculation of relative values. Before and at various time points after surgery, serum levels of parameters shown in Supplementary Table 6 were determined. Cystatin C was measured on the Cobas 8000 system (Roche®) (Supplementary Table 6). A schematic overview of the experimental timeline of the study is shown in Supplementary Figure 6. Processing of the blood samples was done as described previously [15].

### Kidney transplant recipients

Before and after the dietary intervention, the following data were obtained: bodyweight, age, gender, length, and systolic blood pressure. Before and at various time points after surgery, serum values of parameters shown in Supplementary Table 6 were determined. All patients underwent renal scintigraphy (MAG3) on POD1 to assess renal perfusion. In case of a gradual increase of activity in the parenchyma of the transplanted kidney without evidence of cortical excretion, acute tubular necrosis (ATN) was suspected and recorded. Delayed graft function (DGF) was defined as the need for dialysis in the first postoperative week [37]. Slow graft function (SGF) was defined as an estimated glomerular filtration rate (eGFR) ≤10 ml/min/1.73m^2^ at day six after transplantation [20]. Surgical complications were graded using the Clavien-Dindo classification [38].

### mRNA-sequence analysis

For gene expression analysis, kidney biopsies of 10 donors per group were used. Biopsies were put in 2 mL Eppendorf tubes (Eppendorf Group, New York, USA) containing 1 mL of RNA*later* RNA Stabilization Reagent (QIAGEN Benelux B.V., Venlo, the Netherlands), and were kept at 4°C for at least 48 hours. Total RNA extraction and measurement of RNA concentration was done as described previously [12, 13]. The RNA quality was expressed as the RNA integrity number and values ranged between 6.1 and 8.2. mRNA polyA-affinity purification and subsequent Ion Proton (Thermo Fisher) next-generation sequencing was performed by the Microarray Department of the University of Amsterdam, the Netherlands. Handling, analysis and visualization of the data were performed in R (R foundation). Principal component analysis and density plots of the raw counts revealed an outlying sample that had a very low RNA concentration, which was omitted from further analysis. After filtering, 175,756 transcripts were analyzed. Differentially expressed transcripts between diet types within gender were calculated using DESeq2. False Discovery Rate correction was performed as described by Storey and Tibshirani [39, 40]. Complete raw and normalized microarray data and their MIAME compliant metadata have been deposited at the Gene expression Omnibus (GEO) database GSE103532 (https://www.ncbi.nlm.nih.gov/geo/).

### Statistical analysis

Categorical data are presented as numbers and continuous variables as median ± interquartile range (IQR). The data were tested for normality using the Shapiro–Wilks test and subsequent visual assessment. Continuous data were compared using the non-parametric Mann–Whitney U test. A Bonferroni correction for multiple testing was performed on the postoperative kidney function parameters. In the living kidney donors, this correction was done separately for the short-term outcome until POD3 and the outcome until POMo3. In the transplant recipients, the cutoff for short-term outcome was set at POD7; the cutoff for outcome when kidney function has stabilized, was set on POMo6. To assess changes in eGFR and creatinine concentrations over time for both donors and recipients, lme4 [41] for R 3.3.3 was used to perform a linear mixed effects analysis of the relationship between the outcomes of interest and treatment category. Time was modelled as a factor variable as the relationship between the time passed between observation moments is not linear, the correlation with outcome is not known to be linear and we also wanted to model the preoperative measurement. In order to compute the difference, the treatment groups on each time point, the mixed effects model was fitted without an intercept. POD-1 values were not included in the models. In addition, data were adjusted for age and/or gender. The analyses were performed using Statistical Packages for Social Sciences 23.0 (IBM Inc., Chicago, IL, USA), R (R foundation), GraphPad Prism (GraphPad Software Inc., version 5.01), and Office Excel (Microsoft (Office) 2016).

## Supplementary Material

Supplementary Figures

Supplementary Tables

Supplementary Data 1
