# Supplementary material for: Protein and calorie restriction may improve outcomes in living kidney donors and kidney transplant recipients
Source: Aging (Albany NY). 2020 Jul 11;12(13):12441–67. doi: 10.18632/aging.103619 (PMC7377854; doi:10.18632/aging.103619)
Supplement: Supplementary Figures [file aging-12-103619-s003..pdf]

## SUPPLEMENTARY FIGURES

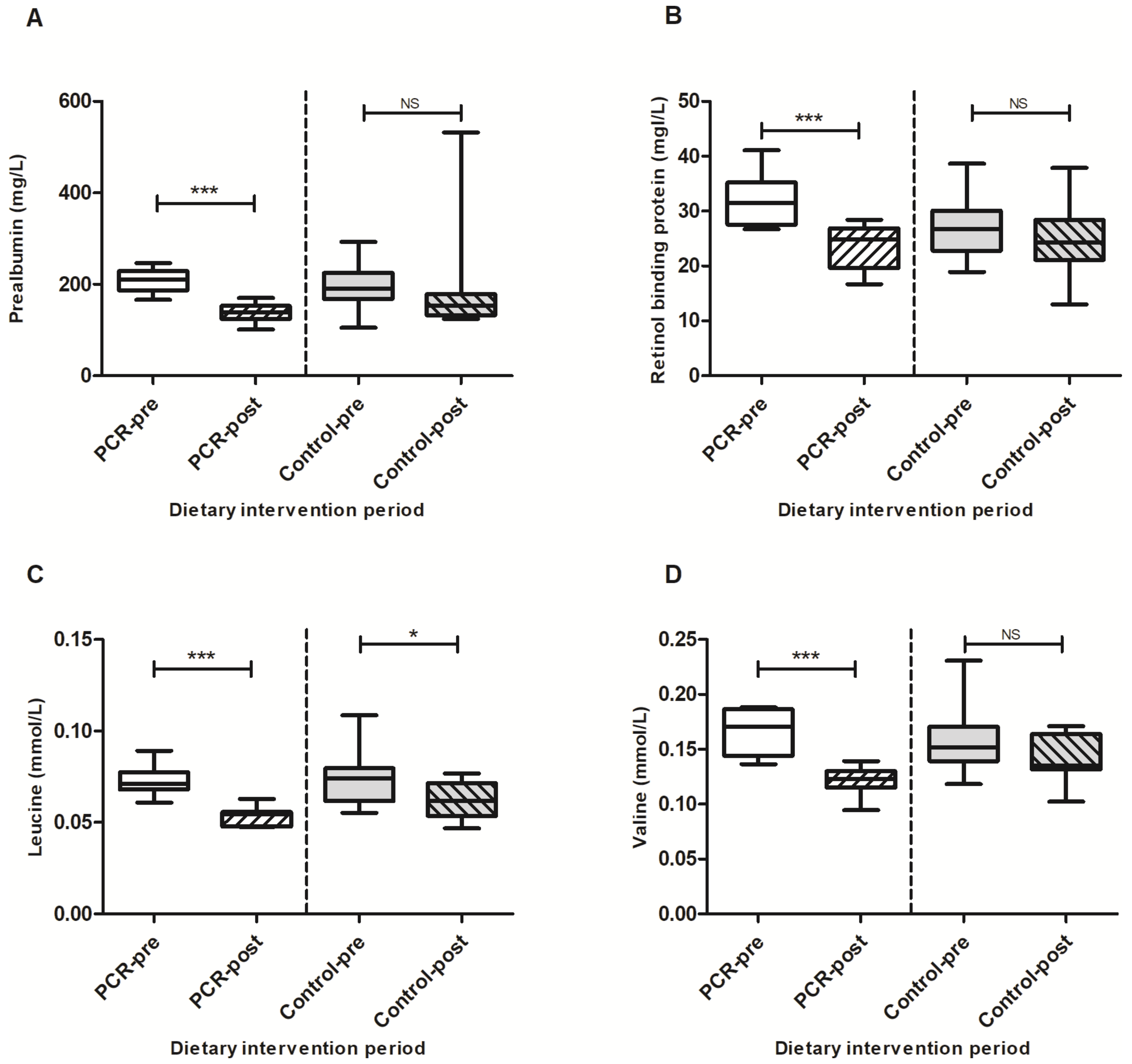


**Supplementary Figure 1. Compliance markers in living kidney donors prior to surgery.** Compliance markers in living kidney donors before and after the PCR diet compared to the control group. (**A**) Levels of prealbumin were significantly decreased after the PCR diet compared to the control group. (**B**) Retinol binding protein levels were also significantly decreased after the PCR diet. (**C**) Absolute levels of the branched-chain amino acid leucine were decreased after the PCR diet. (**D**) The absolute numbers of valine were also significantly decreased after the PCR diet. Values are depicted as mean 95% confidence interval. * = P < 0.05; *** = P < 0.001; NS = not significant. PCR = protein and caloric dietary restriction.

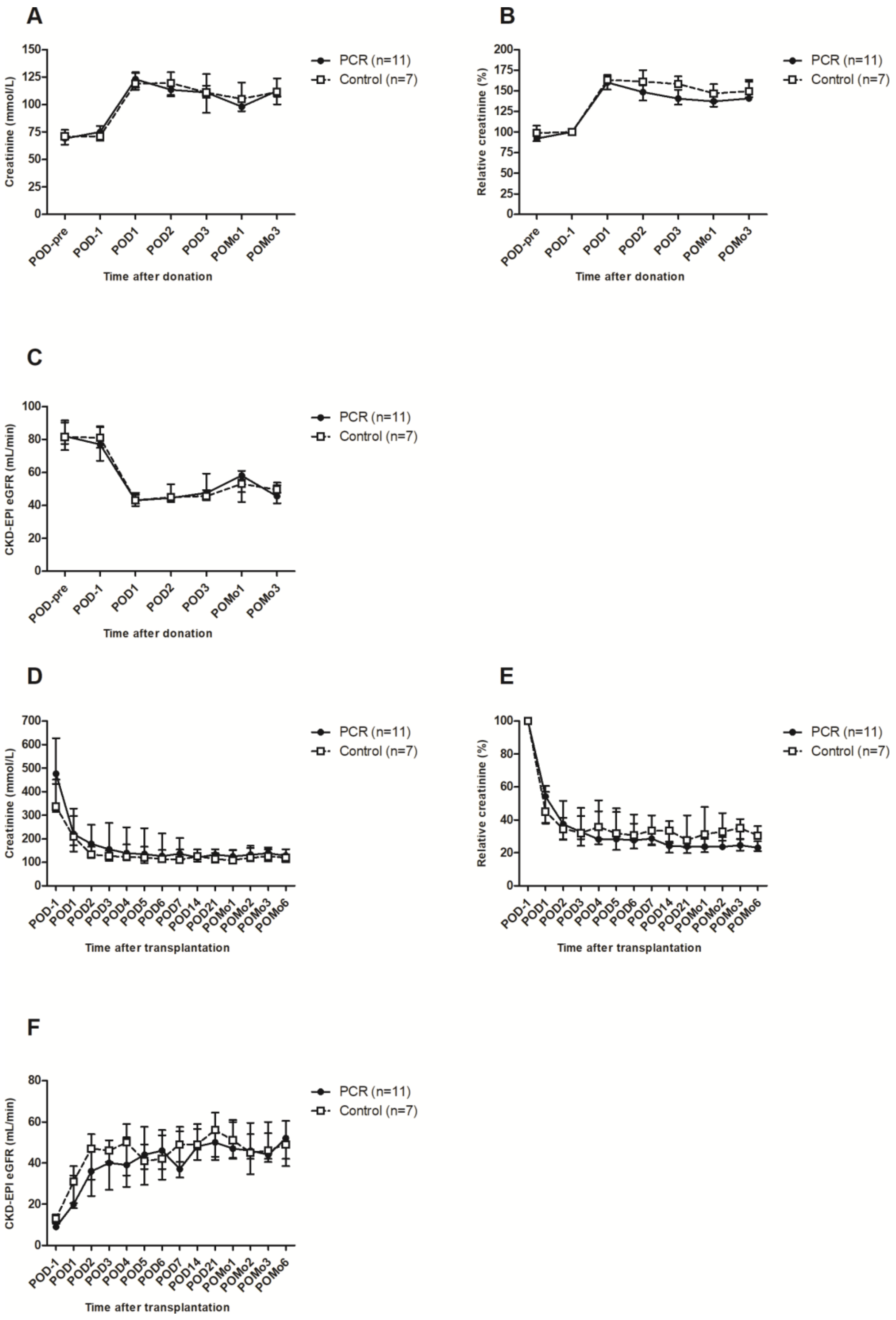


**Supplementary Figure 2. Kidney function of all female kidney donors and kidney transplant recipients prior to and after kidney donation.** (**A**) Absolute levels of serum creatinine of the female kidney donors did not differ between the two intervention groups, while the (**B**) Relative creatinine levels showed a trend towards improvement in the PCR diet group in the first postoperative days. (**C**) Estimated glomerular filtration rate (eGFR) showed a similar result, with no changes in absolute eGFR levels. In the kidney transplant recipients of female living kidney donors, no significant changes were seen in the (**D**) absolute creatinine level, the (**E**) relative creatinine levels, and the absolute eGFR (**F**). Values are depicted as median ± interquartile range. PCR = protein and caloric dietary restriction; POD = postoperative day; POMo = postoperative month; eGFR = estimated glomerular filtration rate. *= P<0.05.

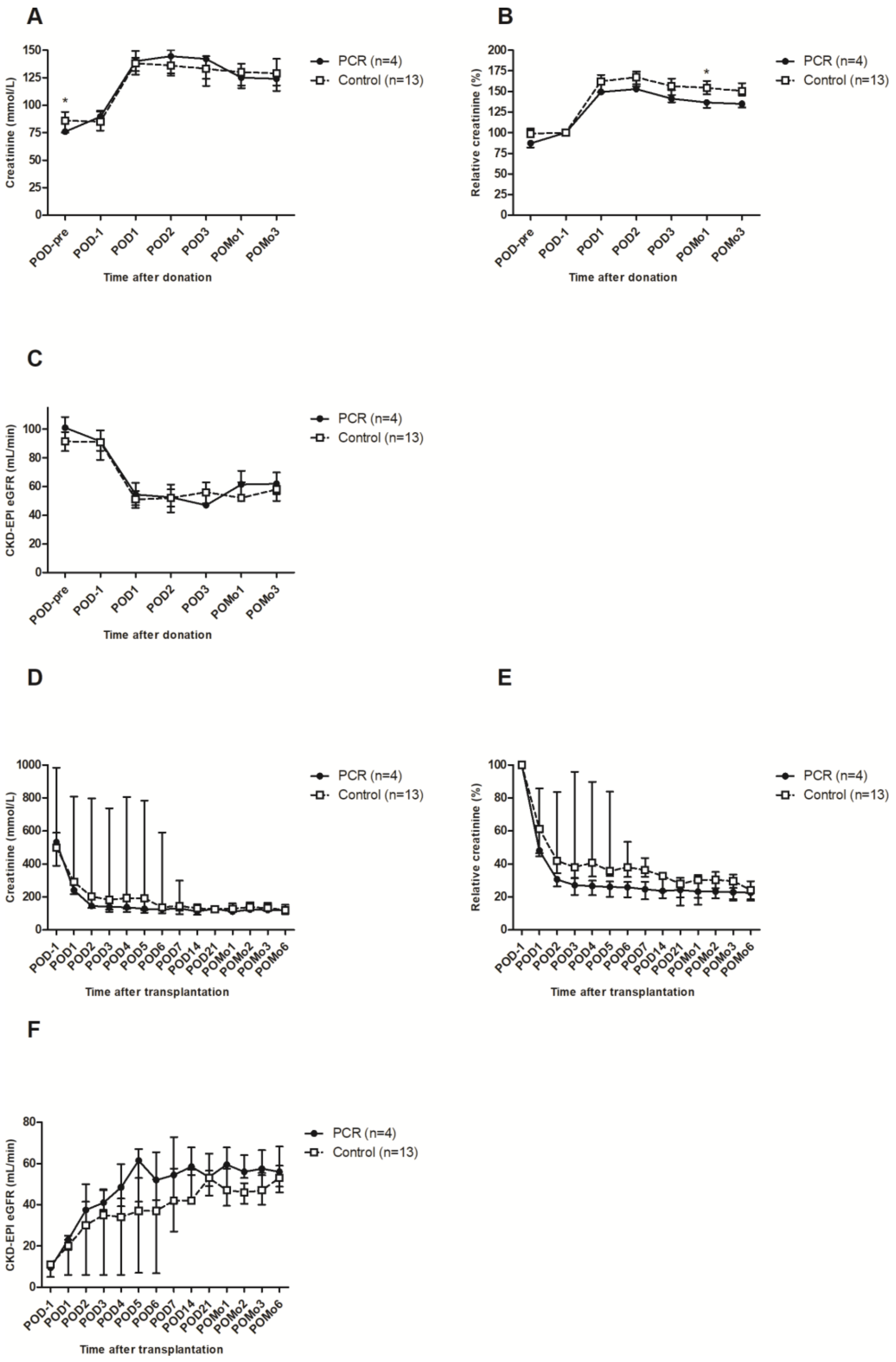


**Supplementary Figure 3. Kidney function of all male kidney donors and kidney transplant recipients prior to and after kidney donation**. (**A**) Absolute levels of serum creatinine of the male kidney donors were significantly higher prior to start of the PCR diet, while no differences between the two intervention groups were seen postoperatively. (**B**) Relative creatinine levels were significantly improved in the PCR diet on postoperative month (POMo)1. (**C**) Estimated glomerular filtration rate (eGFR) showed a lower eGFR in the PCR prior to start intervention and no differences postoperatively. In the kidney transplant recipients of male kidney donors, (**D**) absolute creatinine levels were improved as from POD1 until POD7, while (**E**) relative creatinine levels were better as from POD3 until POD14. (**F**) Absolute eGFR showed a trend towards improvement postoperatively. Values are depicted as median ± interquartile range. PCR = protein and caloric dietary restriction; POD = postoperative day; POMo = postoperative month; eGFR = estimated glomerular filtration rate. *= $P<0.05$.

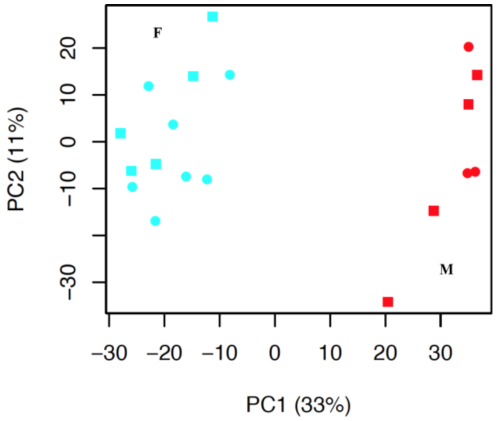


**Supplementary Figure 4. Principal component analysis plot of female and male kidney tissue.** An unbiased principal component analysis of all kidney tissue samples obtained during kidney transplantation revealed a strong separation based on gender on both the principal component axis (PC) 1 and 2. Principal component axis 1 is depicted on the x-axis, while component axis 2 is depicted on the y-axis. Biopsies from the protein and caloric dietary restriction diet kidneys are depicted with squares, while biopsies from control kidneys are depicted with circles. F = female; M = male.

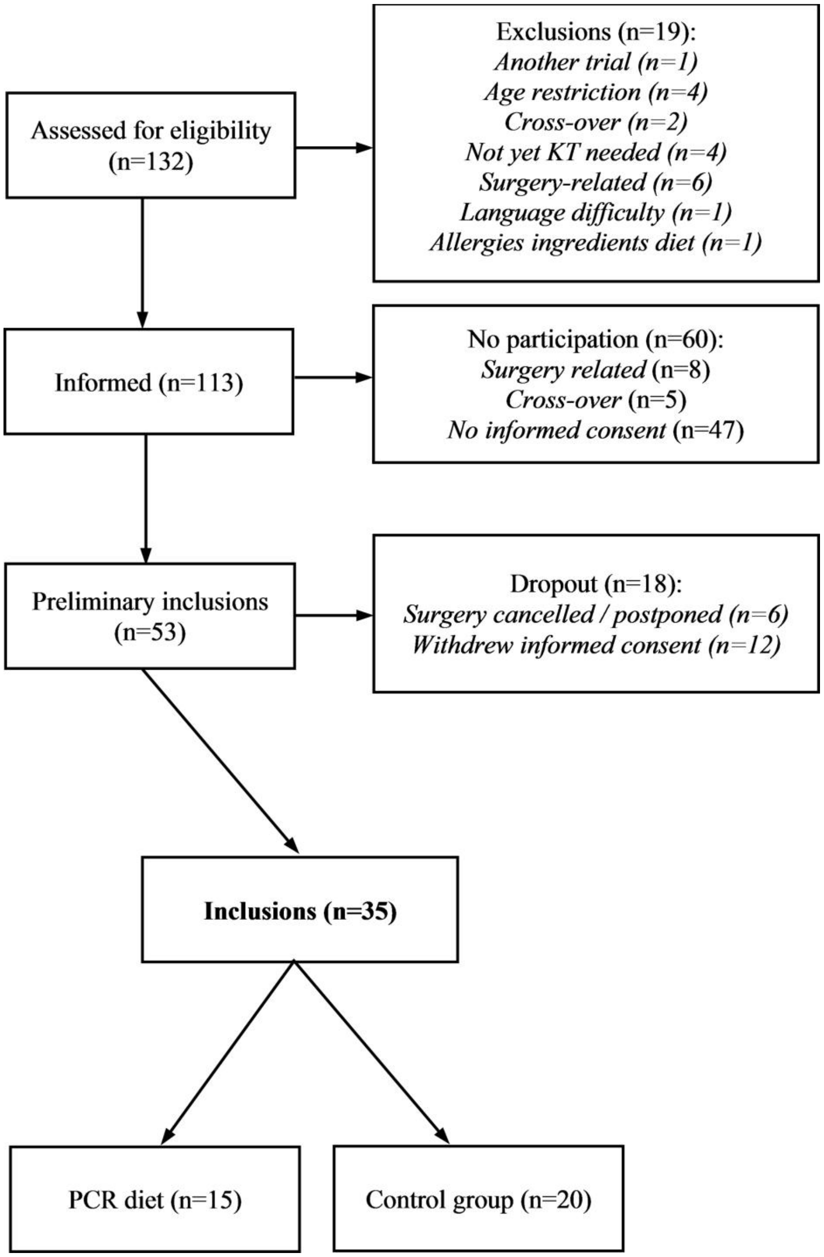


**Supplementary Figure 5. Flowchart of the inclusions and exclusions of the living kidney donors.** Exclusions were based on preset exclusion criteria. Eligible kidney donors were approached at the outpatient clinic at the Erasmus MC, University Medical Center Rotterdam, the Netherlands. After information about the study was given, 57% of eligible donors refrained from participating in the study. Cross-over = cross-over program at which the surgery takes place outside of the Erasmus MC. KT = kidney transplantation. PCR = protein and caloric dietary restriction.

**Donors**

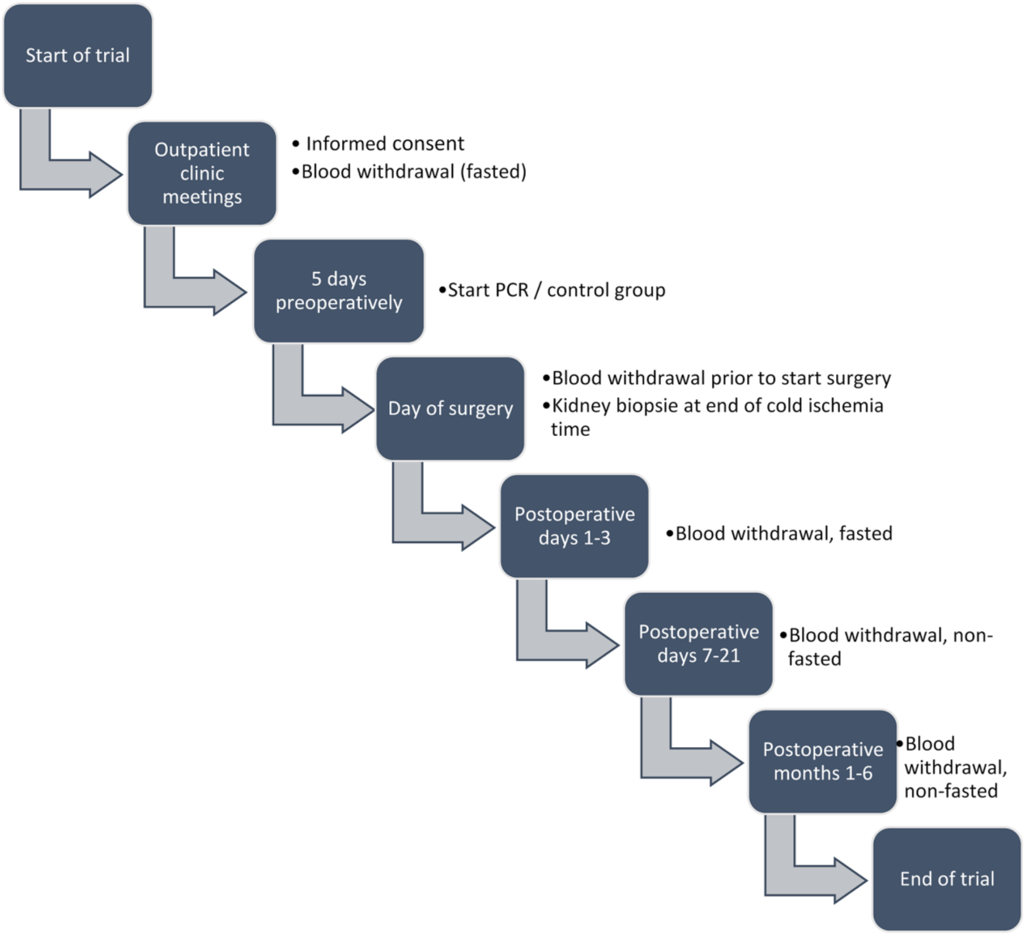


**Recipients**

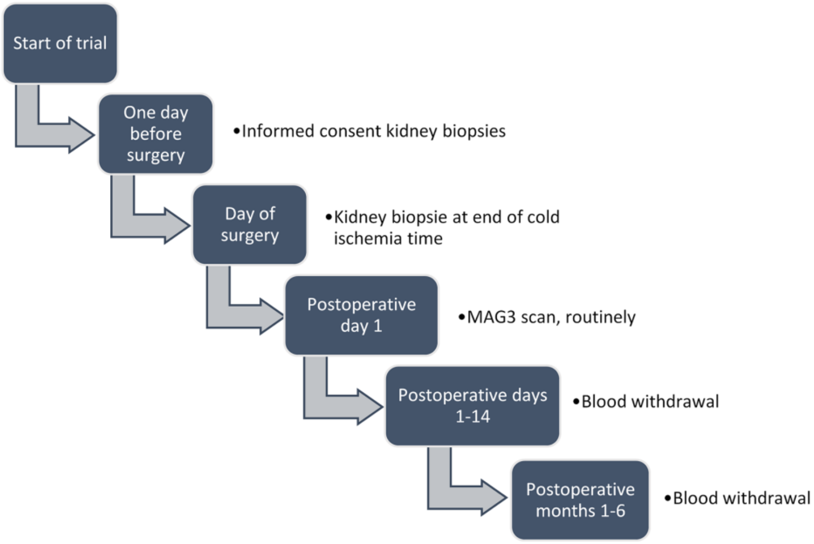


**Supplementary Figure 6. Timeline of living kidney donors and recipients participating in the study, from start until the end of the trial.**
