# Supplementary material for: Protein and calorie restriction may improve outcomes in living kidney donors and kidney transplant recipients
Source: Aging (Albany NY). 2020 Jul 11;12(13):12441–67. doi: 10.18632/aging.103619 (PMC7377854; doi:10.18632/aging.103619)
Supplement: Supplementary Tables [file aging-12-103619-s002..pdf]

SUPPLEMENTARY TABLES

Supplementary Table 1. Perioperative outcome of donors and recipients.

A. Donors			
**Parameter**	**PCR (n=15)**	**Control (n=20)**	**P-value**
Duration of surgery (HH:MM)	03:29 ± 00:10	03:53 ± 00:10	0.11
Start surgery (HH:MM)	08:11 ± 00:05	08:18 ± 00:07	0.43
Blood loss (milliliters)	47 ± 17	82 ± 24	0.24
Duration warm ischemia time 1 (MM:SS))	03:20 ± 00:18	03:23 ± 00:17	0.90
Duration cold ischemia time (HH:MM)	02:37 ± 00:10	02:45 ± 00:12	0.65
Duration of warm ischemia time 2 (MM:SS)	18:47 ± 01:12	20:00 ± 00:48	0.45
Complications during surgery (yes/no)	1/15	0/20	0.33

B. Recipients			
**Parameter**	**PCR (n=15)**	**Control (n=20)**	**P-value**
Duration of surgery (HH:MM)	03:08 ± 00:11	03:06 ± 00:13	0.92
Start of surgery (HH:MM)	11:54 ± 00:13	12:18 ± 00:10	0.17
Time of kidney perfusion (HH:MM)	13:41 ± 00:16	14:06 ± 00:15	0.31
Time of biopsy (HH:MM)	13:20 ± 00:17	13:28 ± 00:11	0.77
Blood loss (milliliters)	180 ± 30	231 ± 41	0.33
Urine production during surgery (yes/no)	1/15	5/20	0.14
Complications during surgery (yes/no)	1/15	0/20	0.33

Parameters are depicted as mean ± standard error of the mean. PCR = protein and caloric dietary restriction. HH:MM = hours:minutes. MM:SS = minutes:seconds. P-value < 0.05 is considered significant.

Supplementary Table 2. Random intercept mixed-effects differences, standard errors (Std. Error) and *P*-values for kidney function outcome of the living kidney donors and their kidney transplant recipients.

A. Living kidney donors

Absolute values

Parameter / Time point	Creatinine (μmol/L)			CKD-EPI eGFR (mL/min)		
	Difference	Std. Error	*P*-value	Difference	Std. Error	*P*-value
POD-pre	- 1,83	6,09	0,765	- 6,68	3,66	0,074
POD1	- 6,57	6,09	0,286	- 2,82	3,66	0,445
POD2	- 14,02	6,13	**0,027**	+ 0,38	3,67	0,918
POD3	- 15,35	6,29	**0,018**	+ 1,22	3,78	0,748
POMo1	- 14,52	6,19	**0,023**	+ 1,73	3,70	0,643
POMo3	- 10,23	6,13	0,102	- 1,42	3,68	0,701

Relative values

Parameter / Time point	Relative creatinine (%)		
	Difference	Std. Error	*P*-value
POD1	- 4,38	72,44	0,399
POD2	- 13,95	76,15	**0,010**
POD3	- 15,53	88,28	**0,006**
POMo1	- 14,35	80,27	**0,009**
POMo3	- 9,28	77,61	0,084

B. Kidney transplant recipients

Absolute values

Parameter / Time point	Creatinine (μmol/L)			CKD-EPI eGFR (mL/min)		
	Difference	Std. Error	*P*-value	Difference	Std. Error	*P*-value
POD-pre	- 23,43	85,23	0,784	- 1,15	5,969	0,848
POD1	- 161,60	85,23	0,062	+ 1,10	5,969	0,854
POD2	- 188,02	85,23	**0,031**	+ 2,37	5,969	0,693
POD3	- 199,73	85,23	**0,022**	+ 6,50	5,969	0,280
POD4	- 202,02	85,23	**0,021**	+ 8,82	5,969	0,144
POD5	- 185,74	85,60	**0,033**	+ 11,28	5,969	0,063
POD6	- 140,75	85,23	0,103	+ 7,46	5,995	0,218
POD7	- 142,53	85,62	0,100	+ 5,98	5,996	0,322
POD14	- 84,16	86,04	0,331	+ 7,94	6,027	0,192
POD21	- 52,44	86,50	0,546	+ 3,46	6,027	0,568
POMo1	- 59,44	86,50	0,494	+ 8,44	6,027	0,166
POMo2	- 50,75	86,50	0,559	+ 7,16	6,027	0,239
POMo3	- 50,74	86,50	0,559	+ 5,05	6,027	0,405
POMo6	- 53,36	86,04	0,537	+ 2,85	6,027	0,638

Relative values			
Parameter / Time point	Relative creatinine (%)		
	Difference	Std. Error	*P*-value
POD1	- 21,82	10,46	**0,040**
POD2	- 28,63	10,46	**0,007**
POD3	- 28,82	10,46	**0,007**
POD4	- 28,47	10,46	**0,008**
POD5	- 21,33	10,46	**0,044**
POD6	- 15,90	10,46	0,132
POD7	- 16,71	10,52	0,116
POD14	- 12,13	10,59	0,255
POD21	- 8,50	10,67	0,428
POMo1	- 9,94	10,67	0,354
POMo2	- 9,52	10,67	0,374
POMo3	- 9,20	10,67	0,391
POMo6	- 6,10	10,67	0,569

CKD-EPI eGFR = Chronic Kidney Disease Epidemiology Collaboration estimated Glomerular Filtration Rate. Differences are based on the outcomes of the CCPR group *versus* the control group. Relative values are set with POD-pre as baseline value, and therefore this time point was not included in the analysis of the relative differences. Significant *P*-values are depicted in bold.

Supplementary Table 3. Baseline characteristics of living kidney donors included in the transciptome analysis.

Parameter	PCR (n=10)	Control (n=10)	P-value
Age (years)	59 (53-61)	51 (44-64)	0.19
Gender (Male/Female)	3/7	5/5	0.39
BMI (kg/m^2)	25.6 (23.3-28.0)	25.4 (23.4-26.0)	0.59
Systolic blood pressure (mm/Hg)	130 (124-136)	128 (124-136)	0.89
Creatinine (mmol/L)	74 (68-82)	78 (71-90)	0.15
eGFR (mL/min)	83 (66-90)	75 (72-83)	0.68
Urea (mmol/L)	5.2 (4.8-5.6)	5.2 (4.0-5.4)	0.46
Glucose (mmol/L)	5.3 (5.1-5.7)	5.1 (4.8-5.8)	0.56
Albumin (g/L)	47 (45-48)	45 (45-47)	0.85
Triglycerides (mmol/L)	1.2 (0.9-1.8)	1.3 (1.2-1.5)	0.62
Hemoglobin (mmol/L)	8.7 (8.4-9.1)	8.9 (8.6-9.2)	0.63
Trombocytes (10^9/L)	241 (222-302)	223 (206-289)	0.48
CRP (mg/L)	1.4 (1.0-2.0)	1.3 (0.8-2.4)	0.30
Leukocytes (10^9/L)	6.5 (5.6-7.6)	5.9 (5.5-6.9)	1.00
Bilirubin (μmol/L)	8.0 (5.3-10.0)	6.0 (5.0-7.3)	0.31
Potassium (mmol/L)	4.5 (4.3-4.6)1	4.4 (4.1-4.8)	0.86
Type of donation (R/U/A)	3/1/6	1/6/3	0.79
Side of nephrectomy (Left/Right)	6/4	6.4	1.00
Method used (Laparoscopic/HARP)	7/3	7/3	1.00

No significant differences (P<0.05) were seen in the baseline characteristics of both groups. BMI = body mass index; eGFR = estimated glomerular filtration rate using the CKD-EPI formula; CRP = C-reactive protein. Type of donation: R = related; U = unrelated; A = anonymous. HARP = hand-assisted retroperitoneal nephrectomy. PCR = protein and caloric dietary restriction.

Supplementary Table 4. Pathway and upstream transcription factor analysis in renal tissue of male donors.

Ingenuity Canonical Pathways	Ratio	Up/Down	P-value	Z-score
Xenobiotic Metabolism Signaling	10/287 (3.5%)	4/6	4.80E-06	N/A
Antigen Presentation Pathway	4/38 (10.5%)	1/3	5.83E-05	N/A
Serotonin Degradation	5/75 (6.7%)	0/5	6.23E-05	N/A
LPS/IL-1-Mediated Inhibition of RXR Function	7/221 (3.2%)	2/5	2.42E-04	N/A
Nicotine Degradation II	4/63 (6.3%)	1/3	4.23E-04	N/A
Superpathway of Melatonin Degradation	4/68 (5.9%)	0/4	5.66E-04	N/A
Allograft Rejection Signaling	4/84 (4.8%)	1/3	1.25E-03	N/A
Thyroid Hormone Metabolism II	3/41 (7.3%)	0/3	1.54E-03	N/A
OX40 Signaling Pathway	4/91 (4.4%)	1/3	1.68E-03	N/A
Autoimmune Thyroid Disease Signaling	3/47 (6.4%)	1/2	2.28E-03	N/A
Graft-versus-Host Disease Signaling	3/48 (6.2%)	1/2	2.43E-03	N/A
Phenylalanine Degradation IV	2/14 (14.3%)	0/2	2.68E-03	N/A
Nicotine Degradation III	3/54 (5.6%)	0/3	3.40E-03	N/A
Type I Diabetes Mellitus Signaling	4/111 (3.6%)	2/2	3.46E-03	N/A
Melatonin Degradation I	3/63 (4.8%)	0/3	5.25E-03	N/A

The top 15 overrepresented pathways derived from the differentially expressed transcripts (DET) in the protein and caloric dietary restriction (PCR) diet compared to the control group in kidney biopsies of male donors. The pathways show their corresponding ratio of regulated genes as percentage of total genes in the pathway, the P-value and the Z-score for predicted activation of inhibition of the pathways.

Upstream regulator	Description	Z-score	P-value	Gene log ratio
**KLF4**	Kruppel like factor 4	**+2.198**	**7.17E-04**	**-0.304**
**NUPR1**	Nuclear protein 1, transcription regulator	**+2.000**	**3.39 E-01**	**+0.081**
RELA	RELA proto-oncogene, NF-κB subunit	+1.970	2.53 E-03	-0.513
KLF5	Kruppel like factor 5	+1.957	2.78 E-04	+0.803
CCND1	Cyclin D1	+1.387	1.37 E-02	+0.294
JUN	Jun proto-oncogene, AP-1 transcription factor subunit	+1.296	4.75 E-02	-0.129
HIF1A	Hypoxia inducible factor 1 alpha subunit	+1.250	5.40 E-04	+0.763
MYC	MYCN proto-oncogene, bHLH transcription factor	+1.224	1.97 E-04	+0.537
NOTCH1	Notch 1	+1.188	1.49 E-02	+0.357
TWIST1	Twist family bHLH transcription factor 1	+1.000	2.04 E-03	-0.152
TP73	Tumor protein 73	+0.639	7.73 E-04	+0.170
NKX2-3	NK2 homeobox 3	+0.447	9.09 E-03	+0.039
SMARCA4	SWI/SNF related, matrix associated, actin dependent regulator of chromatin, subfamily a, member 4	+0.378	2.59 E-02	-0.350
NFKBIA	NFκB inhibitor alpha	+0.295	4.07 E-03	+0.163
GATA3	GATA binding protein 3	+0.152	3.62 E-02	-0.181
TP53	Tumor protein 53	+0.128	1.99 E-06	-0.259
TP63	Tumor protein 63	+0.101	7.14 E-03	+0.116
SP1	Sp1 transcription factor	-0.063	1.50 E-03	-0.240
ATF3	Activating transcription factor 3	-0.106	1.8e E-03	-0.182
NFE2L2	Nuclear factor, erythroid 2 like 2	-0.562	3.28 E-02	+0.331
CEBPA	CCAAT/enhancer binding protein alpha	-0.727	3.54 E-03	+0.441
MYCN	MYCN proto-oncogene, bHLH transcription factor	-0.923	2.36 E-02	-0.030
HMGA1	High mobility group AT-hook 1	-1.000	1.13 E-02	+0.945
CREBBP	CREB binding protein	-1.004	8.07 E-03	+0.409
KLF3	Kruppel like factor 3	-1.134	3.35 E-03	-0.302
CEBPB	CCAAT/enhancer binding protein beta	-1.353	2.65 E-03	-0.186
**HNF4A**	Hepatocyte nuclear factor 4 alpha	**-3.331**	**7.37 E-04**	**-0.470**

Differentially regulated upstream transcription factors (TFs) derived from the DET in the protein and caloric dietary restriction (PCR) diet compared to the control group in kidney biopsies of male donors, with their corresponding Z-score and gene log ratio. Significantly activated or inhibited TFs are depicted in bold.

Supplementary Table 5. Composition and energy content of the protein and caloric dietary restriction (PCR) diet.

Diet<br>Average content per 100 gram/mL	PCR diet
Energy (kcal)	507
(kJ)	2120
Protein (g)	4.98
Protein (% of energy intake)	4.0
Casein (g)	4.3
Whey protein (g)	0.4
Carbohydrates (g)	67.0
Carbohydrates (% of energy intake)	53.0
Glucose (g)	0.8
Fructose (g)	0.0
Lactose (g)	3.2
Maltose (g)	2.5
Sucrose (g)	14.0
Polysaccharides (g)	46.3
Other (g)	0.2
Fat (g)	24.5
Fat (% of energy intake)	43
Saturated fat (g)	7.7
Monounsaturated fat (g)	7.3
Polyunsaturated fat (g)	9.4
Linoleic acid (g)	8.1
A-linoleic acid (g)	0.9
Fibres	0.0
Sodium (mg)	128
Potassium (mg)	272
Chloride (mg)	163
Calcium (mg)	85
Phosphor (mg)	147
Magnesium (mg)	30
Ferritin (mg)	0.0
Zinc (mg)	0.0
Copper (mg)	0.0
Manganic (mg)	0.0
Fluoride (mg)	0.0
Molybdenum (µg)	0.0
Selenium (µg)	0.0
Chrome (µg)	0.0
Iodine (µg)	0.0
Vitamin A (µg-RE)	0.0
Carotenoids (mg)	0.0
Vitamin D (µg)	0.0
Vitamin E (mg α-TE)	0.0
Vitamin K (µg)	0.0
Thiamin (mg)	0.0
Riboflavin (mg)	0.0
Niacin (mg NE)	0.0
Panthotheenzuur (mg)	0.0
Vitamin B6 (mg)	0.0
Foliumzuur (µg)	0.0
B12 (µg)	0.0
Biotin (µg)	0.0
Vitamin C (mg)	0.0
Choline (mg)	0.0

Supplementary Table 6. Serum parameters measured in live kidney donors at various time points during the study.

Parameter	Assay performed at	Time points of measurements
Creatinine (mmol/L)	Hospital laboratory	Outpatient clinic, daily during hospital admission, POMo 1, 3 & 6
eGFR (mL/min)	Hospital laboratory	Outpatient clinic, daily during hospital admission, POMo 1, 3 & 6
Urea (mmol/L)	Hospital laboratory	Outpatient clinic, daily during hospital admission, POMo 3 & 6
Glucose (mmol/L)	Hospital laboratory	Outpatient clinic, daily during hospital admission, POMo 1, 3 & 6
Albumin (g/L)	Hospital laboratory	Outpatient clinic, daily during hospital admission, POMo 1, 3 & 6
Triglycerides (mmol/L)	Hospital laboratory	Outpatient clinic, daily during hospital admission, POMo 1, 3 & 6
Hemoglobin (mmol/L)	Hospital laboratory	Outpatient clinic, daily during hospital admission, POMo 1, 3 & 6
Trombocytes (10^9/L)	Hospital laboratory	Outpatient clinic, daily during hospital admission, POMo 1, 3 & 6
CRP (mg/L)	Hospital laboratory	Outpatient clinic, daily during hospital admission
Leukocytes (10^9/L)	Hospital laboratory	Outpatient clinic, daily during hospital admission, POMo 1, 3 & 6
Bilirubin (µmol/L)	Hospital laboratory	Outpatient clinic, daily during hospital admission
Potassium (mmol/L)	Hospital laboratory	Outpatient clinic, daily during hospital admission, POMo 1, 3 & 6
Prealbumin (mg/L)	Beckman	Outpatient clinic, peroperatively
Retinol binding protein (mmol/L)	Diazyme	Outpatient clinic, peroperatively
Leucine (mmol/L)	Nightingale	Outpatient clinic, peroperatively, POD 1 & 2
Valine (mmol/L)	Nightingale	Outpatient clinic, peroperatively, POD 1 & 2
Cystatin C (CYSC2)	Roche® (Cobas 8000)	Hospital admission, peroperatively, POD 1, 2 & 3 (if available)

eGFR = estimated glomerular filtration rate using the CKD-EPI formula; PoMo = postoperative month; POD = postoperative day; CRP = C-reactive protein.
